# Newcastle disease virus mediated apoptosis and migration inhibition of human oral cancer cells: A probable role of β-catenin and matrix metalloproteinase-7

**DOI:** 10.1038/s41598-019-47244-y

**Published:** 2019-07-26

**Authors:** Sudhir Morla, Ajay Kumar, Sachin Kumar

**Affiliations:** Department of Biosciences and Bioengineering, Indian Institute of Technology Guwahati, Guwahati, Assam 781039 India

**Keywords:** Virus-host interactions, Diseases

## Abstract

Cancer cell metastasis and its dissemination are most enigmatic and challenging aspects in the development of its therapeutics. Newcastle disease virus (NDV) is a well-studied avian paramyxovirus frequently isolated from birds and rarely from mammals. Since the first report of its oncolytic property, many NDV strains were studied for its effect in various cancer cells. In the present study, NDV strain Bareilly was characterized for its apoptotic potential and migration inhibition in human oral cancer cells. The NDV mediated apoptosis was confirmed by flow cytometry, DNA laddering, and immunoblotting. Moreover, NDV decreased the mitochondrial membrane potential suggesting an intrinsic pathway of apoptosis in oral cancer cells. NDV infection in oral cancer cells results in migration inhibition by a reduction in levels of MMP-7. MMP-7 is one of the key target genes of β-catenin. While overexpression of MMP-7 reversed the inhibitory effect of NDV mediated migration suggested its possible involvement. Wnt/β-catenin is an essential pathway for cell growth, differentiation, and metastasis. The involvement of the Wnt/β-catenin pathway in NDV infection has never been reported. Our results showed that NDV dysregulates Wnt/β-catenin by down-regulation of p-Akt and p-GSK3β leading to degradation of β-catenin. Furthermore, NDV infection leads to a reduction in cytoplasmic and nuclear levels of β-catenin. The study will provide us with a better insight into the molecular mechanism of NDV mediated oncolysis and the key cellular partners involved in the process.

## Introduction

Newcastle disease virus (NDV) is a well-studied avian paramyxovirus frequently isolated from birds^1,2^. NDV causes mild to a serious infection in both wild and poultry birds depending on its variant strains^3^. NDV is an enveloped virus containing a single-stranded negative-sense RNA genome and divided into three pathotypes namely, lentogenic (non-lytic), mesogenic (lytic) and velogenic (lytic)^2,4^. Virulent strains of NDV are known for their oncolytic properties because of their effective replication and selective killing of cancer cells^5,6^. Since the first report of their oncolytic property, many NDV strains were studied for their effect in various cancer cells^7,8^. Non-pathogenic nature of NDV to humans and absence of pre-existing immunity are the added advantages of its uses in human medicine. The NDV strain 73T is now under phase II clinical trial by NCI, USA, for the treatment of Melanoma^9^. NDV strains V4UPM, AF2240, D90 were also shown to have oncolytic properties in various cancer cells^10–12^. Tumor cells generally have a defective antiviral response, which supports replication of NDV^13^. Several mechanisms were proposed for NDV mediated apoptosis, which includes both lytic and non-lytic cycles causing enhancement of immune response^14–18^. Development of NDV as a vector to express foreign genes have been explored to enhance its oncolytic activity^19^. Recombinant NDVs expressing cytokines like IL-2, GM-CSF, IFNγ, and TNF-α have shown an increased level of oncolytic activity in various cancer models as compared to its wild-type strain^20,21^.

Matrix metalloproteinases (MMP) are Zn^2+^ dependent proteases, which play a critical role in the degradation of extracellular matrix and cause cancer cell migration^22,23^. MMP-1, -2, -7, -9 and -14 are well studied and known to be elevated in various cancer cells^24,25^. NDV mediated inhibition of cancer cell migration was reported in various cancer cells^26,27^. Although the studies are confined to MMP-2 and -9, its underlying molecular mechanism is not addressed.

Metastasis of cancer cells is the major hurdle in the successful treatment of cancer and the development of its therapeutics. The canonical Wnt/β-catenin pathway plays an important role in cell-cell adhesion, cell communication, and proliferation^28,29^. In the absence of Wnt ligands, β-catenin is regulated by a cytoplasmic complex that includes adenomatous polyposis coli (APC), serine threonine glycogen synthase kinase-3β (GSK-3β) and AXIN_1_^30,31^. The mutation of APC has been implicated in β-catenin mediated carcinogenesis and enhanced cancer cell migration^32,33^. The Wnt and Akt (protein kinase B) canonical regulate phosphorylation of GSK-3β at serine 9 leading to its inactivation^34–36^. Persistent activation of Akt in various types of cancer modulate phosphorylation inactivation of various proapoptotic proteins leading to cancer cell survival and chemoresistance^37,38^. In addition, alteration in Wnt/β-catenin pathway results in translocation of β-catenin to the nucleus resulting in enhanced cell proliferation and aberrant expression of various genes such as; cyclin D1, c-Myc and MMP-7^34,39,40^. APC and β-catenin are considered as potential diagnostic markers for malignant transformation in oral squamous cell carcinoma^41^. MMP-7, a downstream target of β-catenin is involved in metastasis by immunohistochemistry of pancreatic and oral cancers^42^. Previous studies have shown the role of β-catenin/MMP-7 in cancer invasion and progression^24,43^. However, the role of MMP-7 in NDV mediated migration inhibition has not been explored.

The specific purposes of the present study are, to determine the apoptotic potential of NDV strain Bareilly in oral carcinoma cells and to explore the probable molecular mechanism of its migration inhibition. To the best of our knowledge, this is the first report of modulations of MMP-7 and β-catenin upon NDV infection in the cancer cells.

## Results

### Effect on cell viability and apoptosis upon infection with NDV

**Figure 1:**
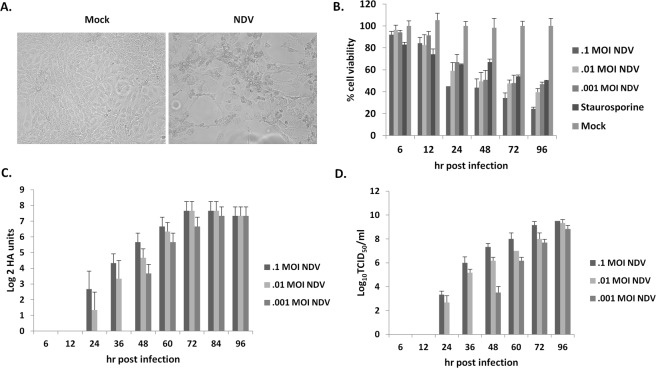
NDV related kinetics and cytotoxicity in SAS cells. The images show cell control, and NDV specific cytopathic effects (**A**). The images were taken at 20X magnification under EVOS FLoid cell imaging station (ThermoFisher Scientific, USA). SAS cells were infected with different MOI and MTT assay was performed at regular time intervals (**B**). Time-course viral growth kinetics were NDV was infected at different MOI and the supernatant was collected for virus titration by HA (**C**) and TCID_50_ method (**D**). MTT, HA, and TCID_50_ data represent the mean ± SD of three independent experiments.

NDV strain Bareilly showed sustained cytopathic effect in human oral squamous cell carcinoma cell line (SAS). The cytopathic effects include rounding and detachment (Fig. 1A). The cytotoxicity of SAS cells following NDV infection was analyzed at different time intervals. The cell survivability was decreased to 60% and 24% following 24 and 96 hr post-infection, respectively (Fig. 1B). On analyzing the cell culture supernatant collected at regular time points, high viral titers were recorded (Fig. 1C,D). Moreover, NDV strain Bareilly also showed cytotoxicity in breast cancer cells (MCF7), human neuroblastoma cells (IMR32), and the cervical cancer cells (HeLa) in a dose-dependent manner (Supplementary Fig. S1).

**Figure 2:**
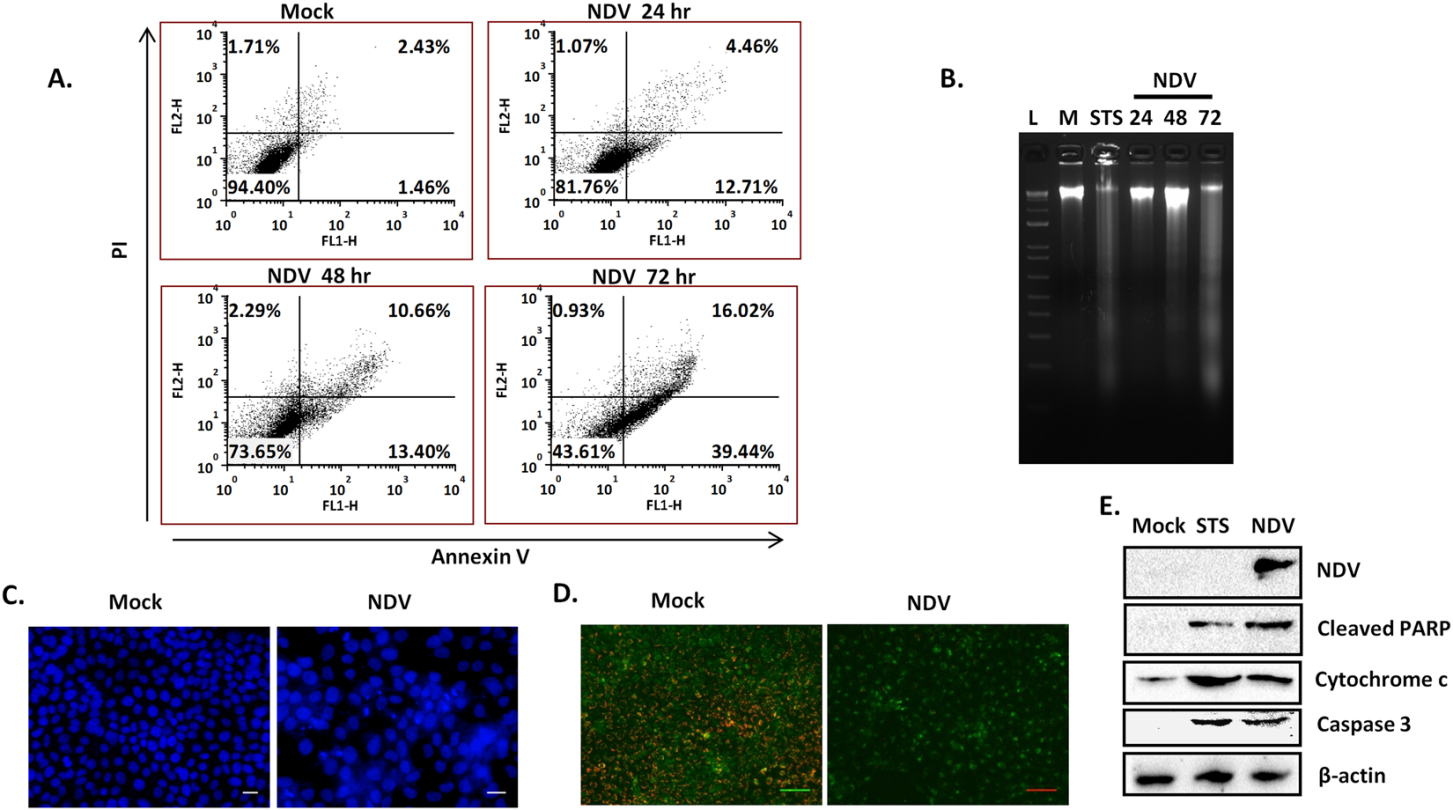
NDV induced apoptosis in SAS cells. Images show analysis of apoptosis by NDV (0.1 MOI) on SAS by Annexin V FITC and PI assay using flow cytometry (**A**). Agarose gel image showing DNA laddering post-NDV infection in SAS cells (B). Hoechst 33342 staining of the cells showing apoptosis-related changes in the nucleus (**C**). Image showing the decreased mitochondrial membrane potential upon NDV infection (**D**). In mock-treated SAS cells, mitochondria aggregated JC-1 dye to give red fluorescence while NDV infected cells showed decreased fluorescence. Western blot analysis of cleaved PARP, cytochrome c, and caspase 3 upon NDV infection showed a band size of 89 kDa, 14 kDa and 17 kDa, respectively. β-actin was used as a loading control for the blots (**E**). “M” stands for the mock control, “L” for DNA ladder and STS for staurosporine.

About 39.4% of SAS cells showed the early stages of apoptosis as compared to 1.4% in control cells when analyzed by flow cytometry at 72 hr post-NDV infection (Fig. 2A). Apoptotic effect was further assessed by Hoechst 33342 staining and DNA laddering. DNA laddering in SAS cells infected with NDV started at 48 hr and was distinct at 72 hr post-infection (Fig. 2B). The mock uninfected cells showed no laddering, while the STS showed positive laddering in SAS cells. On Hoechst 33342 staining, chromatin condensation was readily observed in SAS cells at 48 hr post-infection with NDV (Fig. 2C). The loss of mitochondrial membrane potential was much evident in NDV infected cells by loss of red/green fluorescence intensity of JC-1 dye (Fig. 2D). Furthermore, NDV induced apoptosis in SAS cells was determined by western blot analysis. The increased amount of cytochrome c was observed in NDV infection SAS cells. Similarly, cleavage of PARP and caspase 3 was observed following infection of NDV in SAS cells (Fig. 2E).

### NDV inhibits migration of oral cancer cells

**Figure 3:**
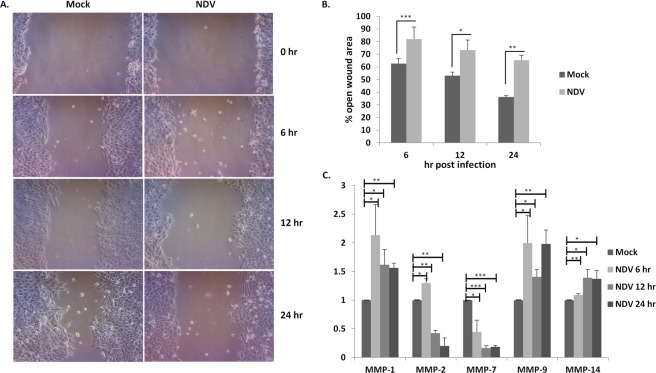
The images show NDV infection in SAS cells leading to migration inhibition. Wound healing assay was performed on SAS cells, the cells were infected with 0.1 MOI NDV and cell migration was evaluated at regular time points post-infection (**A**). The images were taken at 10X magnification under inverted microscope (Lebomed, USA). The open wound area was calculated at every time point by using ImageJ software and converted into a percentage with respect to zero hr (**B**). The mRNA levels of various MMP’s were analyzed 6, 12, 24 hr post-NDV infection, each experiment was repeated thrice using GAPDH as a normalizing control (**C**). Data is represented as fold change upon NDV infection relative to control. qRT-PCR was performed 3 times. T-test using Microsoft Excel, *P < 0.05, **P ≤ 0.01, ***P ≤ 0.001.

Wound healing assay (WHA) was performed to determine the inhibitory effect of NDV on SAS cells. The open wound area was calculated at regular intervals of post-NDV infection in SAS cells. WHA on infection with NDV was suppressed at all-time points (Fig. 3A). Nearly 20% inhibition of migration was observed at 6 and 12 hr post-infection as compared to uninfected SAS cells. Although 20% cell death was observed till 12 hr, a maximum of 30% inhibition was observed 24 hr post-infection as compared to uninfected cells (Fig. 3B). The NDV specific cytopathic effect was visible in the infected SAS cells. The expression of MMPs was analyzed by real-time PCR (Fig. 3C). MMP-1, MMP-9, and MMP-14 showed no significant changes in NDV infection while a reduction in the levels of MMP-2 and MMP-7 was observed. A much significant reduction in MMP-7 was observed in all the time points. However, MMP-2 levels were reduced temporally upon NDV infection.

### Investigation of MMP-7 mediated migration inhibition in oral cancer cells

**Figure 4:**
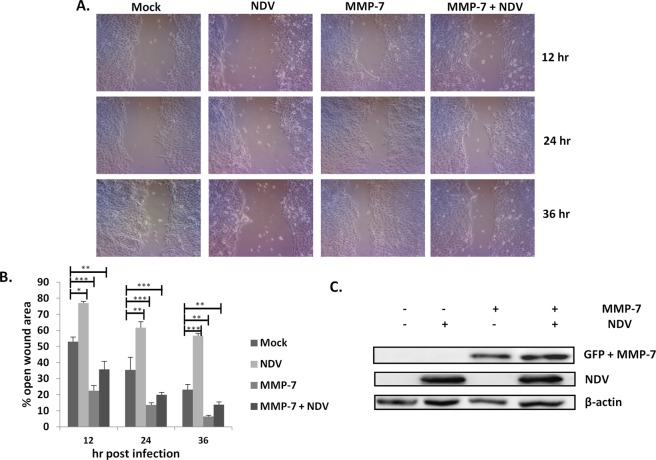
Overexpression of MMP-7 antagonized NDV related migration inhibition (**A**). The images were taken at 10X magnification under inverted microscope (Lebomed, USA). Wound healing assay was performed by overexpressing MMP-7 by transfection of pcDNA3-GFP-MMP-7 (Addgene plasmid # 11989). MMP-7 only, uninfected cell and NDV infected controls were included. The open wound area was quantified at every time point by using ImageJ software (**B**). Western blot showing the overexpression of MMP-7 (GFP tagged) and presence of NDV (**C**). β-actin was used as a loading control.

MMP-7 was overexpressed in the SAS cells and WHA was performed. MMP-7 overexpressing SAS cells were more aggressive as compared to untransfected control cells (Fig. 4A,B). Moreover, MMP-7 overexpression showed no inhibitory effect of NDV on SAS cells migration. The protein expression of MMP-7 was confirmed by western blot analysis (Fig. 4C).

### Role of β-catenin/MMP-7 on NDV mediated inhibition

**Figure 5:**
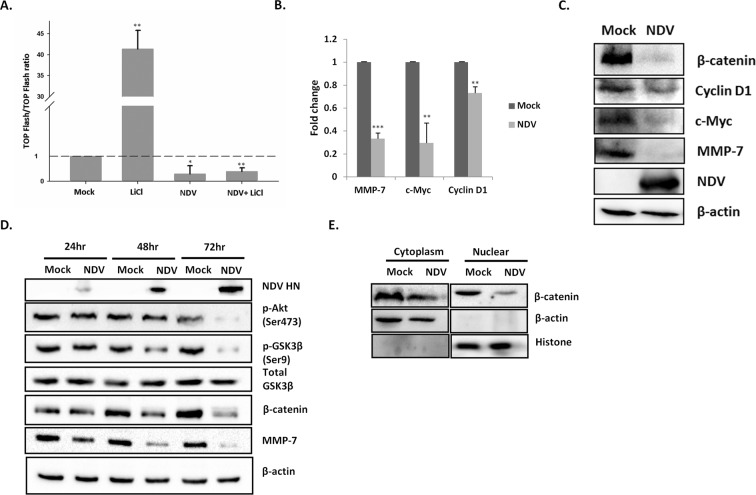
NDV infection reduces β-catenin expression. TOPFlash/FOPFlash luciferase assay was performed in SAS cells infected with NDV (0.1MOI). LiCl at a concentration of 20 mM was used to stimulate the cells along with mock infected and NDV infected cells controls. TOP/FOP ratio is plotted on Y axis and was broken at 3 and restarted at 30 to accommodate the higher values. The dashed line states the basal value corresponding to mock treated samples. The data is average of three independent experiments (**A**). The mRNA levels of various β-catenin regulated genes were analyzed 48 hr post-NDV infection, the experiment was performed at least three times and GAPDH was used as a normalizing control (**B**). Data are represented as fold change upon NDV infection relative to control. Western blots showing the suppression of β-catenin beyond detection and down regulation of β-catenin regulated genes 48 hr post-infection (**C**). SAS cell lysates were collected at 24, 48 and 72 hr post-NDV (0.1MOI) infection and analyzed for p-Akt (Ser473), total GSK-3β, p-GSK-3β (Ser9), β-catenin, and MMP-7 expression by western blot (**D**). The β-actin serves as the loading control. Cytoplasmic and nuclear fractions were collected from SAS cells 48 hr post-NDV infection (0.1MOI) and analyzed for β-catenin by western blot. The β-actin and histone serve as the loading controls (**E**). T-test using Microsoft Excel, *P < 0.05, **P ≤ 0.01, ***P ≤ 0.001.

TOPFlash/FOPFlash luciferase reporter system^44^, was used to demonstrate the β-catenin transcriptional activity in SAS cells. LiCl stimulated mock samples have shown 41 fold increase in luciferase activity compared to mock cells. In contrast, NDV infected SAS cells stimulated with LiCl showed a reduction in luciferase activity (Fig. 5A). The expression level of β-catenin and its target genes were examined in NDV infected SAS cells by quantitative real-time PCR and western blot analysis. NDV infection in SAS cells showed down-regulation of MMP-7 by 0.67 folds. The β-catenin modulated genes such as c-Myc and cyclin D1 were also analyzed to evaluate its specificity. The c-Myc and cyclin D1 showed 0 .71 and 0.27 fold reduction, respectively, relative to control cells (Fig. 5B). As expected in line with mRNA, protein expression of MMP-7, cyclin D1 and c-Myc showed reduction following NDV infection in SAS cells (Fig. 5C). Taken together, the RNA and protein levels demonstrate that the NDV inhibits the transcriptional activity of the β-catenin. The western blot was performed at various time points to decipher the role of NDV infection on β-catenin levels in SAS cells. There was a significant reduction of β-catenin levels at 72 hr post-NDV infection. A similar effect was recorded in other cancer cells, suggesting the involvement of β-catenin in NDV infection (Supplementary Fig. S2). To address the mechanism of NDV mediated β-catenin reduction, levels of p-Akt (Ser473), total GSK-3β, p-GSK-3β (Ser9) and MMP-7 protein levels were also analyzed by western blotting for same time points. NDV infection decreased the levels of p-Akt, p-GSK-3β, and MMP-7 by the time-dependent manner in SAS cells (Fig. 5D). There was no change in total GSK-3β levels on NDV infection. The subcellular levels of β-catenin were analyzed by western blotting of the cytoplasmic and nuclear fractions. Histone and β-actin were used as loading and purity controls. NDV infection reduced the levels of cytoplasmic and nuclear levels of β-catenin (Fig. 5E).

## Discussion

The first report of NDV as an antineoplastic agent was reported in 1965 by Cassel *et al*.^45^. Many studies have been conducted in animal models as well as cancer patients, which demonstrated the oncolytic and immunostimulatory effect of NDV^46–48^. Importantly, humans do not have pre-existing immunity against NDV. Moreover, it is non-pathogenic to humans because of the species barrier. Clinical safety of cancer patients in various studies highlighted NDV as an attractive target for oncolytic therapy^49,50^. Many strains of NDV were analyzed both *in vitro* and *in vivo* to evaluate the efficacy of its oncolytic activity^51,52^.

In the present study, we have evaluated the apoptotic and inhibitory effect of NDV strain Bareilly in oral cancer cells. Furthermore, the NDV strain Bareilly showed an inhibitory effect on a variety of cancer cell lines (Supplementary Fig. S1). NDV strain Bareilly was previously sequenced from our laboratory^53^. The cleavage site of NDV strain Bareilly corresponds to ^112^RRQKR^116^ similar to well-studied oncolytic NDV strain^54^. Presence of virulent cleavage is reported to be one of the predisposing factors for NDV oncolysis^55^. The NDV related cell viability studies in oral cells were dose and time-dependent similar to earlier reported studies^56^. Furthermore, the apoptosis rate analyzed by Annexin V and PI staining 72 hr post-infection showed 39% Annexin V positive compared to control suggesting the cause of death might be apoptosis. Furthermore, nuclear fragmentation, DNA laddering, and nuclear condensation supported our hypothesis of NDV strain Bareilly to be apoptotic. Apoptotic pathway ultimately results in the activation of caspase 3 and PARP, which leads to morphological changes in cells. Activated PARP and cleaved caspase 3 in SAS cells proved that NDV strain Bareilly is apoptotic.

The mechanism by which NDV causes apoptosis is not well understood and many studies have been conducted to establish its underlying molecular mechanism^17^. The lytic and non-lytic pathways are proposed for NDV mediated oncolysis. However, the concepts are not clear and often contradictory^50^. It has been shown that mitochondrial membrane polarity and cytochrome c level could be a marker to decipher the apoptotic pathways^57^. Briefly, loss of mitochondrial membrane potential leads to intrinsic while activation of caspase 8 leads to the extrinsic pathway^58^. Our studies showed, decrease in mitochondrial membrane polarity and elevated cytochrome c level following NDV infection, suggesting the involvement of the intrinsic pathway of apoptosis.

NDV is known to cause inhibition of migration in various types of cancer cells^26,27^. However, the underlying mechanism is not well known. The reduction of MMP-2 and MMP-9 upon NDV infection is reported in the earlier reports. In our study, NDV strain Bareilly has shown migration inhibition in SAS cells. However, there was no significant reduction in the levels of MMP-9. MMP-2 and MMP-7 were down-regulated by NDV infection in SAS cells. Interestingly, MMP-7 was down-regulated even in early time points post-NDV infection. MMP-2 is well studied in the case of NDV related inhibition, however, the role of MMP-7 has not been explored. In addition, overexpression of MMP-7 reversed the effect of NDV mediated cell migration in SAS. MMP-7 is considered to play an important role in invasion and metastasis in a variety of cancer cells, and it is reported to be elevated in SAS^59,60^. It is well characterized that MMP-7 is one of the targets of the Wnt/β-catenin signaling pathway, which plays a significant role in cell proliferation and oncogenesis^61^. Our studies showed down-regulation in the level of MMP-7 and β-catenin upon NDV infection, suggesting their role in the migration inhibition. TOPFlash/FOPFlash luciferase reporter system showed that NDV reduces β-catenin related transcriptional activity. Moreover, genes such as cyclin D1 and c-Myc, which are modulated through β-catenin also showed down-regulation. The effect was evident in both transcription as well as translation level suggesting it to be both regulatory and functional.

In the canonical Wnt pathway, Akt negatively regulates GSK3β by phosphorylation, leading to cytoplasmic and nuclear accumulation of β-catenin and an increase in its transcriptional activity^34,35^. Our results showed that the protein expression of NDV strain Bareilly started at 24 hr post-infection as reported for its other characterized strains^62^. Further our results showed a decrease in the levels of p-Akt and p-GSK-3β upon NDV infection. Earlier reports suggest that NDV infection decreases p-Akt, however, its effect on GSK-3β has not been explored^63^. It has also been reported that GSK-3β regulates β-catenin level in cells^64^. Our study corroborates with the earlier reports suggesting degradation of β-catenin through downregulation of p-Akt and p-GSK-3β.

Our results suggest that NDV negatively regulates β-catenin and MMP-7 in order to potentiate apoptosis and inhibition of cell migration. Our study for the first time reports the involvement of Wnt/β-catenin signaling pathway in NDV mediated oncolysis. It will be interesting to explore the Wnt/β-catenin signaling pathway in an animal model. The results could be taken as a step towards understanding the NDV mediated cell signaling. Although β-catenin seems to play a role in NDV mediated migration inhibition, it is too early to predict it to be the only way a virus regulates the host physiology.

## Methods

### Virus and cells

The velogenic NDV strain Bareilly, sequenced in our laboratory (GenBank accession number KJ577585) was used in the present study^53^. The virus was propagated in 9-day-old specific pathogen free embryonated chicken egg and the titer was calculated by hemagglutination assay (HA) and plaque assay. The plaque-purified virus was stored at −80 °C for further experiments. The SAS, MCF7, IMR32, and HeLa cells were grown in Dulbecco’s modified Eagle’s medium (DMEM) media with 10% fetal bovine serum (GIBCO, USA) and supplemented with an antibiotic-antimycotic solution (GIBCO, USA).

### Apoptosis study in cancer cells

Cell cytotoxicity was measured by MTT assay, briefly, 5 × 10^3^ cells per well were seeded in a 96 well plate. After attachment, the cells were treated with different multiplicity of infections (MOI) by NDV (0.1, 0.01, and 0.001). The spectrophotometer readings were taken at regular intervals following the standard protocol^56^. Staurosporine (STS) at a concentration of 40 nM was used as a positive control in all the experiments. Annexin V labeled with FITC and propidium iodide (PI) (Invitrogen, USA) was used to determine the translocation of phosphatidylserine, and viability of the cells, respectively. A distinct feature of apoptosis is the fragmentation of genomic DNA. The SAS cells were infected with NDV at an MOI of 0.1 and cells were collected at regular interval. The DNA fragmentation assay was performed as described previously^56^. In addition, fragmentation was also determined by staining the infected cells with Hoechst 33342 dye (Invitrogen, USA). Mitochondrial-specific dual fluorescence dye, JC-1 was used to measure the mitochondrial membrane potential. Cells were infected with NDV at an MOI of 0.1 and stained with JC-1 dye following 48 hr post-infection. As reported, the aggregate of JC1 gives red fluorescence while its diffused form gives a green fluorescence^65^.

### Wound healing assay

5 × 10^5^ cells were seeded in 35 mm dish and infected with NDV at an MOI of 0.1. The wound was created using a 200 µl tip, and the cells were washed with PBS to remove detached cells. The wound was observed under a microscope and analyzed for its diameter at different time intervals. ImageJ software was used to analyze the open wound area. The area at zero hr was taken as 100%, and the relative decrease in the area was calculated at respective time points. Uninfected cells were taken as a negative control for all the experiments.

### Real time-PCR

Total RNA was isolated using RNAiso plus reagent (Takara, Japan). RNA was quantified using µ-drop plate reader (Thermo Scientific, USA). 2 µg of total RNA obtained from control and NDV infected cells was reverse transcribed into cDNA using high-capacity cDNA reverse transcription kit (Thermo Scientific, USA). Real-time qRT-PCR was done using PowerUp SYBR™ Green Master Mix (Invitrogen, USA) on QuantStudio 5 Real-Time PCR System (Thermo Scientific, USA). The primer sequences used in the present study are given in Supplementary Table S1.

### Transfection studies

The SAS cells were seeded (5 × 10^5^ per 35 mm dish) with DMEM and transfected with Lipofectamine 2000 (Invitrogen, USA) using 2 µg of pcDNA3-GFP-MMP-7 (Addgene plasmid # 11989). The cells were infected with NDV following 12 hr post-transfection. The uninfected and only transfected cells were used as controls for the experiment. The lysates collected 48 hr post-infection were used for immunoblot analysis.

### Immunoblotting

SAS cells were seeded in 6 well plates and treated with NDV at an MOI of 0.1 for 48 hr. The media was then removed and the whole cell lysate was collected with RIPA lysis buffer containing 1x ProteoGuard EDTA free protease inhibitor cocktail (Clontech, USA). Cell lysates were collected by centrifugation at maximum rpm for 15 minutes at 4 °C and the supernatant was stored at −80 °C till use. Caspase 3, poly ADP ribose polymerase (PARP), cytochrome C, anti-GFP, β-catenin, cyclin D1, c-Myc, MMP-7, p-Akt (Ser473), β-actin (Thermo Scientific, USA), total GSK-3β, p-GSK-3β (Ser9) (Cell Signaling Technology, USA) and histone (H3) (BioBharati Life Sciences, India) antibodies were used to develop the immunoblots. The polyclonal antiserum generated against NDV in SPF chickens was used to detect the viral specific proteins. The β-actin was used as an internal control for all the immunoblots.

### Luciferase reporter assay

SAS cells (10^4^/well) were seeded in 24 well plate and transfected with 400 ng of TOPFlash or FOPFlash plasmids with 40 ng of pRenillaTK using Lipofectamine 2000 (Invitrogen, USA). Both the plasmids are a kind gift from Dr Randall Moon, University of Washington School of Medicine, USA (Addgene plasmids # 12456 and 12457). TOPFlash contains seven TCF/LEF binding sites followed by firefly luciferase gene, were as FOPFlash serves as a control plasmid containing mutated TCF/LEF binding sites^44^. The cells were infected with 0.1MOI of NDV or mock infected six hr post-transfection. The cells were stimulated with 20 mM LiCl or vehicle for 12 hr and cell lysates were collected 24 hr after infection. Dual luciferase kit (Promega, USA) was used to measure the luciferase activity by GloMax® 20/20 single tube Luminometer (Promega, Madison, WI). For each experiment, uninfected and untreated cells were used as controls. TOP/FOP ration was calculated after normalising the values with renilla.

### Nuclear and cytoplasmic fractionation

The SAS cells in 100 mm tissue culture plates were infected with 0.1MOI NDV. At 48 hr monolayer was washed with PBS twice and lysed by adding 300 µl of PBS containing 1% NP-40, 1 mM dithiothreitol and protease inhibitor cocktail (Clonetech, USA) for 10 min incubation room temperature. The lysate was subjected to centrifugation at 4000 × g for 10 min at 4 °C and the supernatant containing the cytoplasmic fractions were collected. The nuclear pellet was washed twice with PBS and lysed using 35 µl of 1% SDS. The β-actin and H3 were used as internal controls to assure fractionate contamination. All the experiments were performed thrice to reproduce the data as much as possible.

### Statistical analysis

The qRT-PCR, immunoblots, and WHA results from three independent experiments were statistically analyzed. The results were analyzed using the t-test (Microsoft Excel), and the statistical significance was set to P < 0.05.

## Supplementary information

Supplementary figure S1, Supplementary figure S2, Supplementary figure S3, Supplementary figure S4, Supplementary table S1.
