# Supplementary material for: Newcastle disease virus mediated apoptosis and migration inhibition of human oral cancer cells: A probable role of β-catenin and matrix metalloproteinase-7
Source: Sci Rep. 2019 Jul 26;9:10882. doi: 10.1038/s41598-019-47244-y (PMC6659693; doi:10.1038/s41598-019-47244-y)
Supplement: Supplementary file 1 — Supplementary figure S1, Supplementary figure S2, Supplementary figure S3, Supplementary figure S4, Supplementary table S1. [file 41598_2019_47244_MOESM1_ESM.pdf]

Newcastle disease virus mediated apoptosis and migration inhibition of human oral cancer cells: A probable role of β-catenin and matrix metalloproteinase-7.

Sudhir Morla[1], Ajay Kumar[1], Sachin Kumar[1]*

[1]Department of Biosciences and Bioengineering, Indian Institute of Technology Guwahati, Guwahati, Assam 781039, India.

* Corresponding author: Phone: (91) 3612582229; Fax: (91) 3612582249

E-mail: sachinku@iitg.ac.in

Supplementary figure S1. NDV related cell cytotoxicity in cancer cell lines. The breast cancer cells, MCF7 (A), the human neuroblastoma cells, IMR32 (B), and the cervical cancer cells, HeLa (C) were used for the cytotoxicity study of the NDV stain Bareilly. The $TCID_{50}$ data represents the mean ±SD of three independent experiments.

(A)

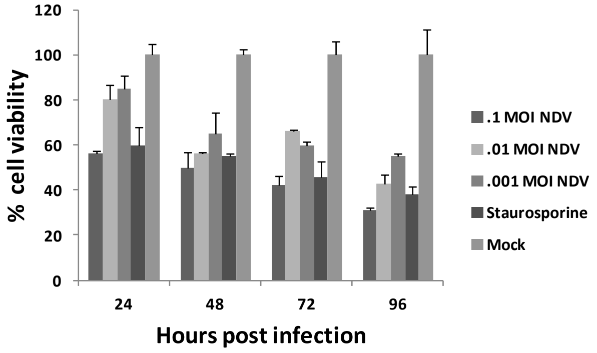

(B)

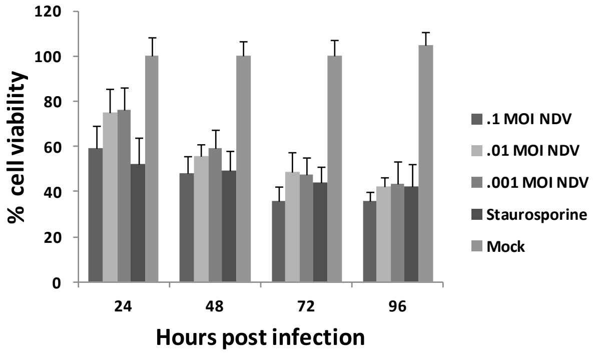

(C)

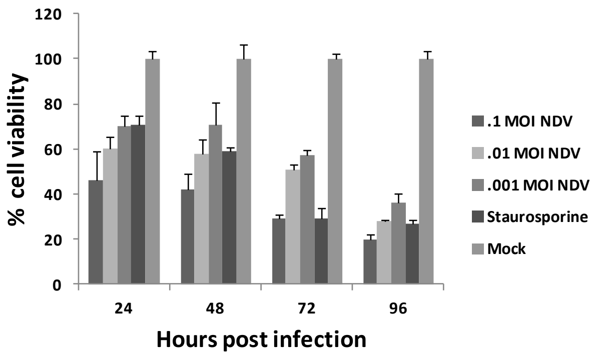

Supplementary figure S2. NDV infection reduced the β-catenin expression in Wnt + MCF7 (A) and T47D (B) breast cancer cells. Cell lysates were collected after infected with NDV (0.1MOI) 48 hr post infection and analyzed for β-catenin levels by western blot. The β-actin serves as the loading control. BR states the biological replicates.

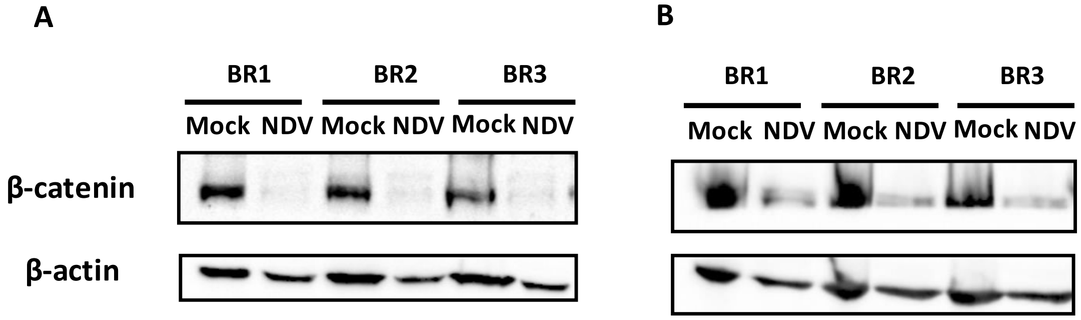

Supplementary figure S3. Original gel images of immunoblots used in figure 4C. Wound healing assay was performed by overexpressing MMP-7 by transfection of pcDNA3-GFP-MMP-7 (Addgene plasmid # 11989) Western blot showing the overexpression of MMP-7(GFP tag) and NDV growth, β-actin was used as loading control. Dotted lines provide an indication of cropped area.

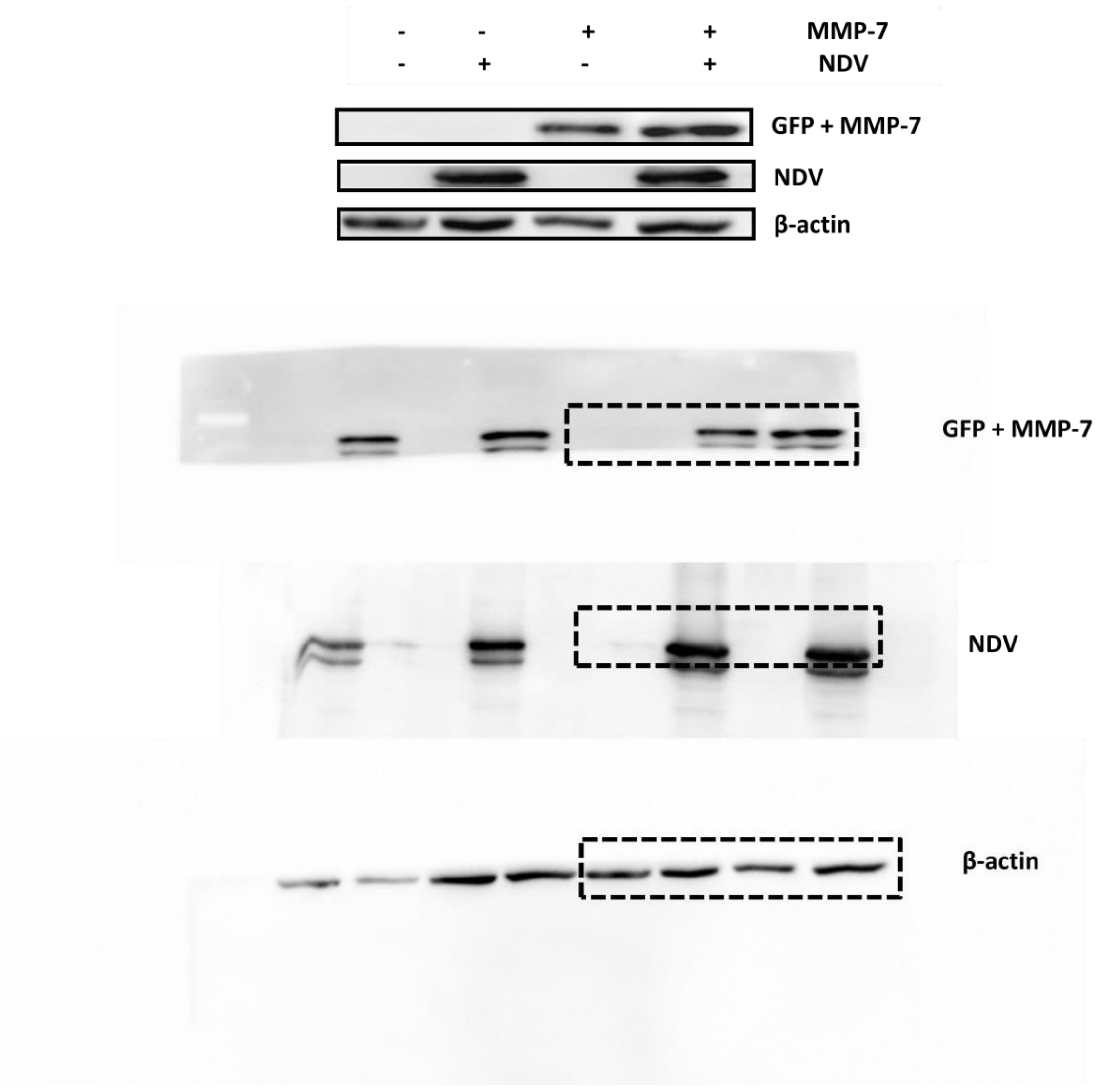

Supplementary figure S4. Original gel images of immunoblots used in figure 5C. Cell lysates were collected of SAS infected with NDV (0.1MOI) at indicated times post infection and analyzed p-Akt (Ser473), total GSK-3β, p-GSK-3β (Ser9), β-catenin, MMP-7 expression by western blot. β-actin serves as loading control. Dotted lines provide an indication of cropped area.

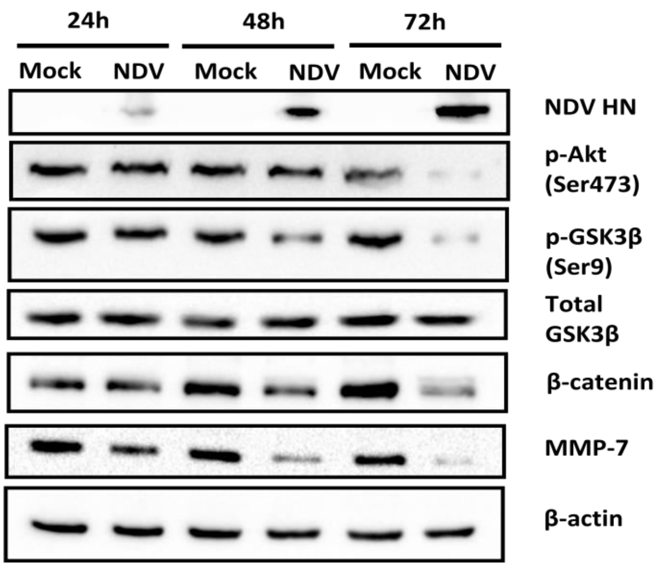

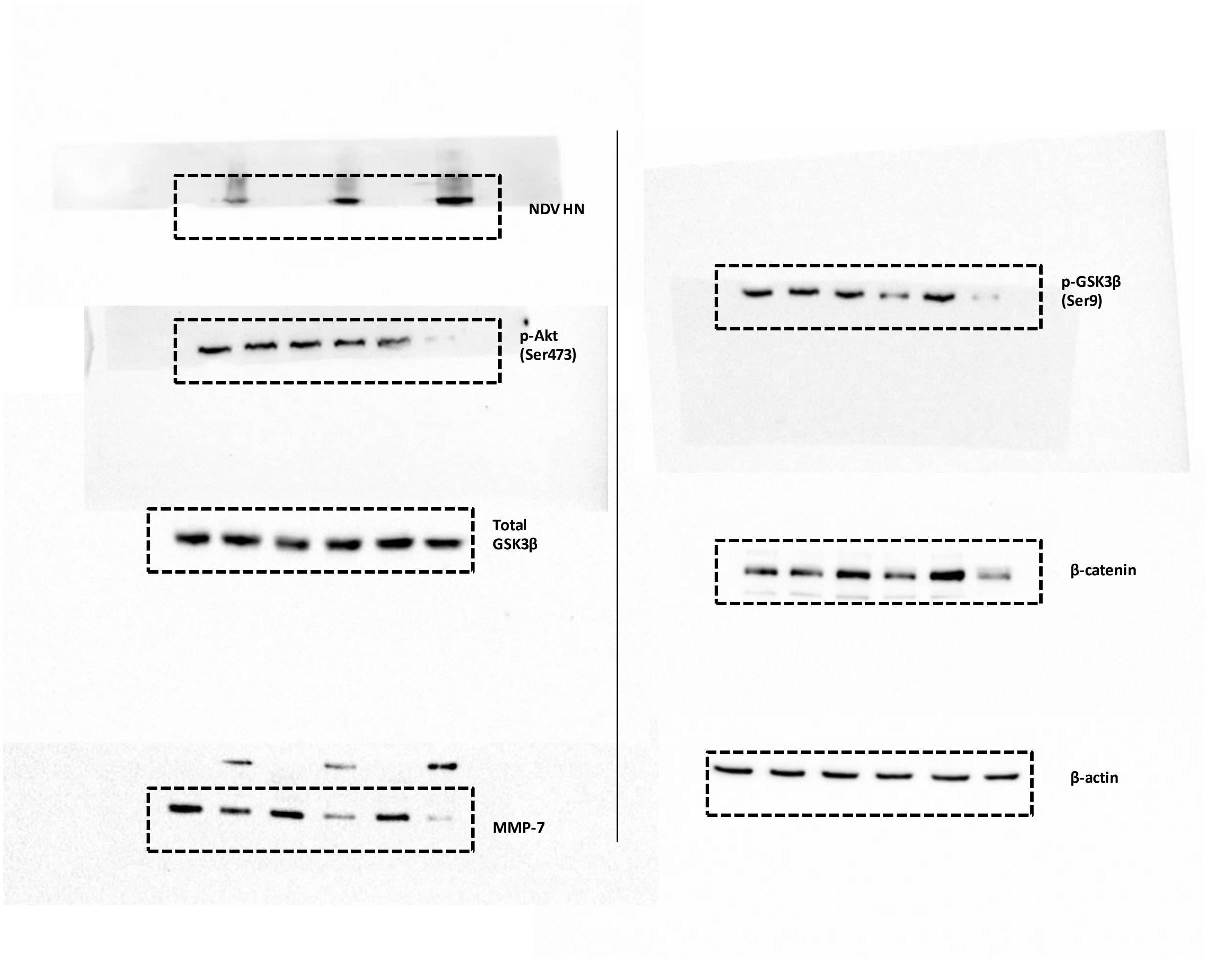

Supplementary table S1. The nucleotide sequences of the primers used for the qRT-PCR analysis of the cells infected with NDV strain Bareilly.

**Primer name**	**Primer sequence (5'-3')**	**Amplicon length (bp)**	**Annealing temperature (^{0}C)**
GAPDH forward	ATGGAGAAGGCTGGGGCTCA	189	60
GAPDH reverse	GTTGTCATGGATGACCTTGGC		
Cyclin D1 forward	GCCCCAACAACTTCCTGTCC	178	60
Cyclin D1 reverse	TCCTCCTCTTCCTCCTCCTC		
B-catenin forward	CAGGGTGCCATTCCACGAC	143	60
B-catenin reverse	AGGGCTCCGGTACAACCTTC		
c-Myc forward	CCGTCCTCGGATTCTCTGCT	231	60
c-Myc reverse	TGGGCTGTCAGGAGGTTTGC		
MMP-1 forward	CAGGGGAGATCATCGGGACA	84	60
MMP-1 reverse	CCAATACCTGGGCCTGGTTG		
MMP-2 forward	CCAAAACGGACAAAGAGTTGGC	131	60
MMP-2 reverse	TGTCTGGGGCAGTCCAAAGAA		
MMP-7 forward	GTTGTATGGGGAACTGCTGAC	157	60
MMP-7 reverse	TCCAGCGTTCATCCTCATCG		
MMP-9 forward	GGAGGCGCTCATGTACCCTA	99	60
MMP-9 reverse	TCAGGGCGAGGACCATAGAG		
MMP-14 forward	GGATCCCTGAGTCTCCCAGA	117	60
MMP-14 reverse	AGCCCGGTTCTACCTTCAGC		
